# Imaging Findings of an Epidermoid Cyst Undergoing Malignant Transformation

**DOI:** 10.5334/jbr-btr.829

**Published:** 2015-09-15

**Authors:** D. Chourmouzi, E. Papadopoulou, G. Karkavelas, A. Drevelegas

**Affiliations:** 1 Department of Diagnostic Radiology, Interbalcan Medical Centre, Thessaloniki, Greece; 2 Department of Pathology, Aristotle University of Thessaloniki, School of Medicine, Thessoloniki, Greece; 3 Department of Diagnostic Radiology, AHEPA University Hospital, School of Medicine, Thessoloniki, Greece

**Keywords:** Epidermoid

## Abstract

Malignant transformation of epidermoid cyst into squamous cell carcinoma (SCC) is rare. We report the case of a 39-year-old woman presenting with dizziness and cerebellar ataxia. MR scan revealed a mass in the left cerebropontine angle compressing the brainstem and the cerebellum, with two main components, a cystic and a solid one. The cystic component displayed imaging findings consistent with an epidermoid cyst. The solid component showed dense calcifications, low signal intensity on T1W, T2W and DW images and peripheral nodular enhancement. MR spectroscopy detected high lipid/lactate peaks and choline/creatine ratio. Imaging findings raised suspicion for malignant transformation, which was confirmed by histopathologic examination revealing an SCC. MR imaging with intravenous administration of gadolinium, DW images and MR spectroscopy can play a critical role in the diagnosis of malignant transformation of an epidermoid cyst.

Malignant transformation of intracranial epidermoid cysts (IECs) is very rare. Clinical deterioration and contrast enhancement of a known IEC or at the surgical site of a removed IEC should raise suspicion for malignant transformation. There are very few reports and to our knowledge no reviews describing the spectral pattern of IEC undergone malignant transformation. We describe a rare case of initial malignant transformation of an IEC, with special emphasis on the spectroscopic findings. MR spectroscopy (MRS) can contribute towards the correct diagnosis.

## Case report

A 39-year-old woman was admitted to our hospital presenting with dizziness and cerebellar ataxia.

**Figure 1:**
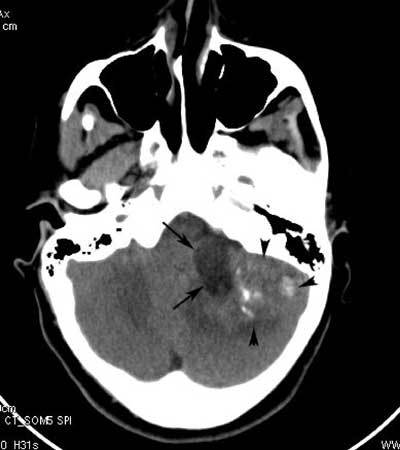
CT scan displays a mass in the left CPA with a cystic (arrows) and a solid component (arrowheads). The solid component of the mass shows dense calcifications.

Computed tomography (CT) scan revealed a large partially cystic mass in the left cerebropontine angle (CPA) with calcifications, compressing the brainstem and the cerebellum (Fig. 1).

**Figure 2:**
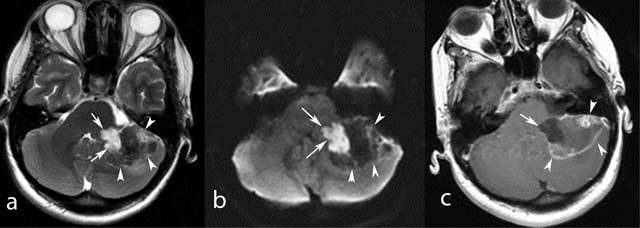
MR scan shows that the cystic component of the mass (arrows) is hyperintense on T2W (a), and DW images (b) with no enhancement on contrast-enhanced T1W images (c), findings compatible with an epidermoid cyst. On the other hand the solid component (arrowheads) has very low signal intensity on T2Wimages (a), and low signal intensity on DW images (b) and peripheral, linear and nodular enhancement on T1W post-contrast-enhanced images (c).

**Figure 3:**
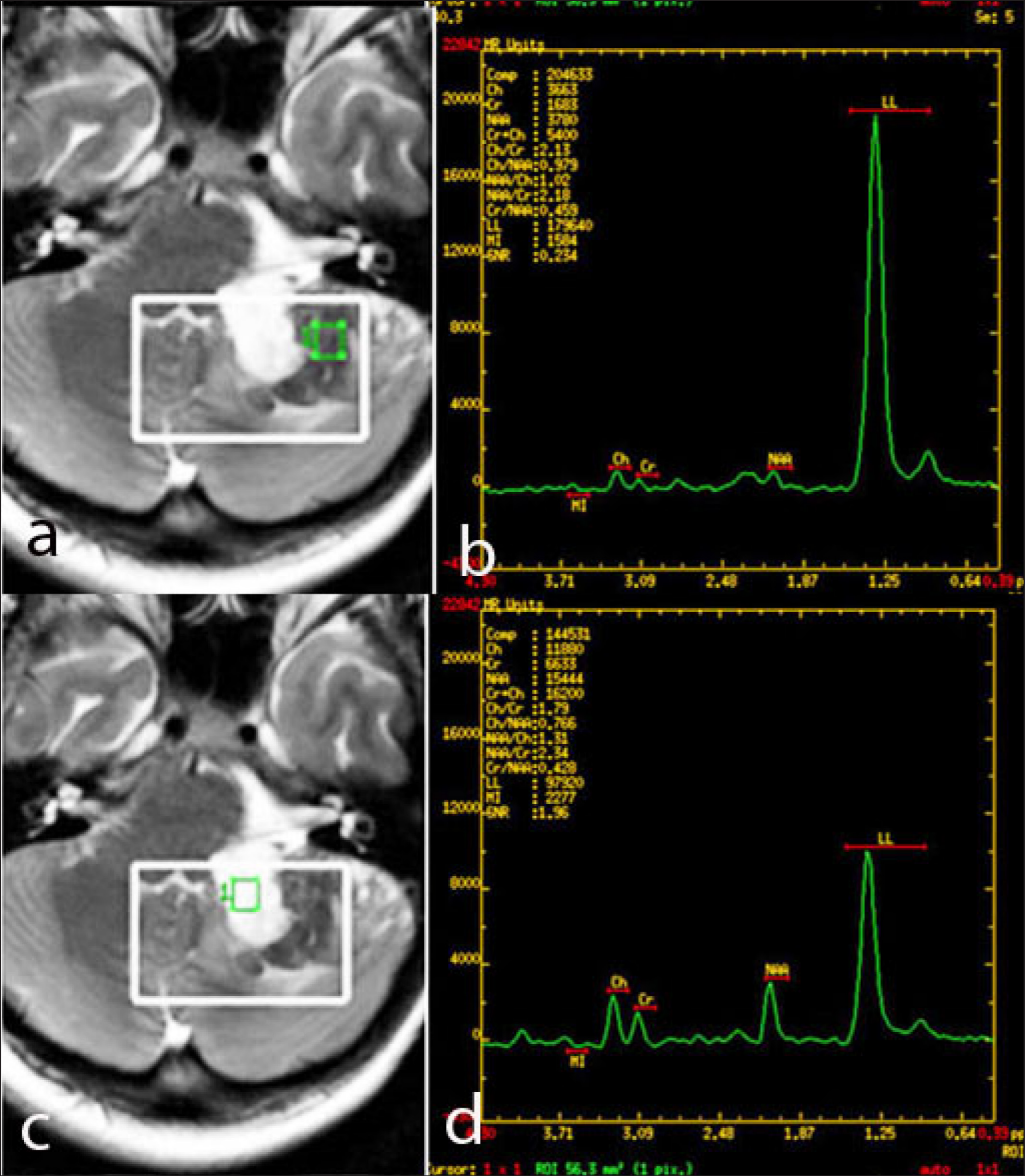
MR Spectroscopy detects high lipid/lactate peak in the solid component of the mass (a, b), lower NAA and relatively higher Cho/Cr ratios than its cystic counterpart (c, d), findings suspicious of malignant transformation.

MR imaging (Figs. 2, 3) confirmed the presence of a large mass in the left CPA. The mass had two components, cystic and solid. The cystic component of the mass was located in the left CPA, extending anteriorly to the 7^th^ cranial nerve and medially compressing the brainstem. It showed low signal intensity on T1W and high signal intensity on T2W ones and with no enhancement after intravenous administration of contrast media. On DW images the mass displayed high signal intensity, ADC 0.8 and the spectral pattern showed a medium lipid/lactate peak at 1.3 ppm, findings typical for IEC. The juxtaposed solid component of the mass compressed the cerebellum causing perilesional edema. It showed low signal intensity on T1W and T2W images. After intravenous administration of contrast media, it displayed heterogeneous, mainly peripherical and in some sites nodular enhancement. DW imaging showed low signal intensity inside the mass and ADC values of 1.34. MRS revealed a high lipid/lactate peak, Choline/Creatine ratio (Cho/Cr) 2.13 and Choline/N-Acetylaspartate ratio (Cho/NAA) 0.766.

**Figure 4:**
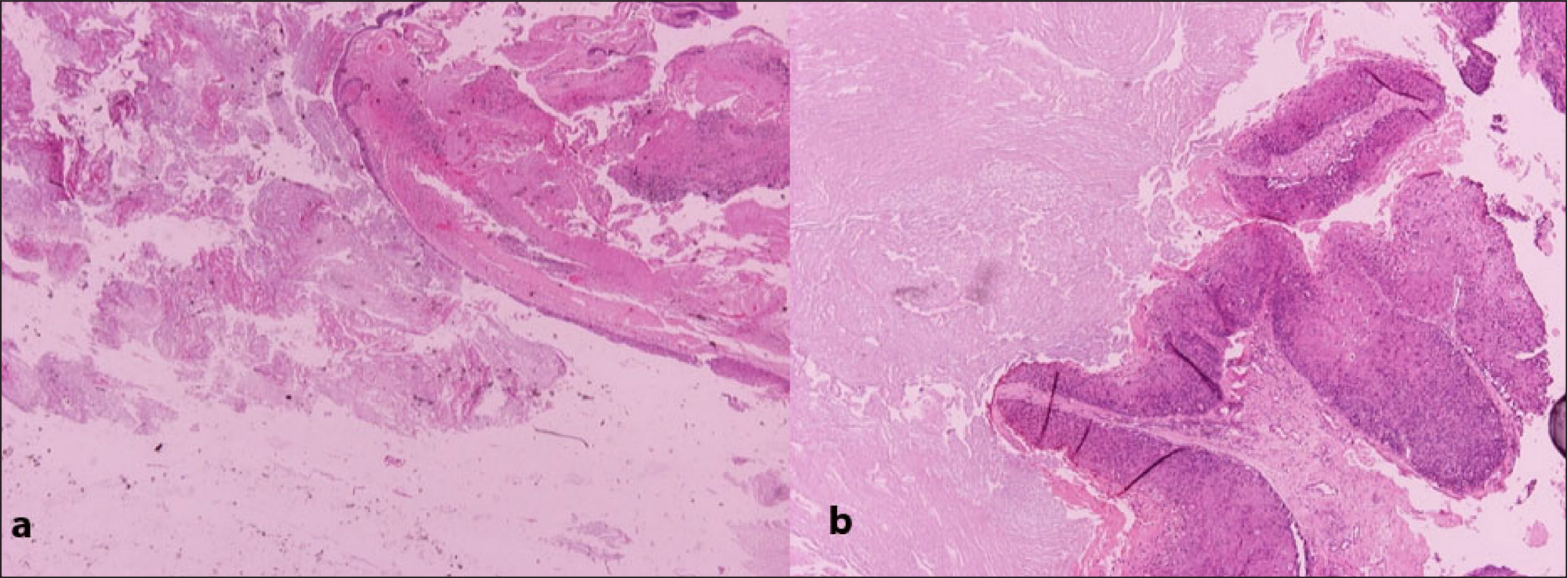
Photomicrographs of (a) the cystic component showing features consistent with benign epidermoid cyst and (b) the solid component showing squamous epithelium with pleomorphic cellularity and high mitotic activity compatible with squamous cell carcinoma.

Surgery was planned and the mass was completely resected. Histopathologic analysis (Fig. 4) revealed that the cystic component was consistent with benign IEC. The adjacent solid component consisted of squamous epithelial cells, with nuclear pleomorphism and mitotic activity. The superficially lined cells were filled with laminated keratin. There were also islands of squamous epithelium with an infiltrative growth pattern.

## Discussion

IECs are rare lesions, accounting for 0,2–1,8% of all intracranial tumors and may arise from retained ectodermal embryonic tissue in the neural groove at 3^rd^–5^th^ week of gestation.

Malignant transformation of IECs is very rare. It is postulated that chronic inflammation caused by repeated microruptures of the cysts or iatrogenic irritation after resection of the cysts may trigger malignant transformation of IECs [1].

Criteria for diagnosis of malignant transformation of an intracranial epithelial cyst according to Garcia [2] and Hamlat [3] are: a) tumor restricted to the intracranial-intradural compartment, b) no extension beyond the dura, cranial orifices, or connection with the middle ear, air sinuses or sella turcica, c) no evidence of nasopharyngeal tumor, d) presence of benign squamous cell epithelium within the malignant tumor and e) metastasis of carcinoma excluded.

According to Hamlat, malignant transormation or intracranial epithelial cysts can be classified into 5 groups: a) ­initial malignant transformation of a benign cyst, b) malignant transformation from remnant cyst, c) malignant transformation of a dermoid and epithelial cyst, d) malignant transformation with leptomeningeal carcinomatosis and e) other malignancies arising from benign cysts.

Our case fulfilled all the criteria of Garcia and Hamlat, and according to Hamlat’s classification, it was characterized as initial transformation of an IEC.

Rapid deterioration of symptoms is the most important clinical indication of malignant transformation. Our patient complained of dizziness and cerebellar ataxia.

MR imaging of IEC is well known, displaying low signal intensity on T1W and high signal intensity on T2W, FLAIR and DW images. Rim enhancement can be detected in 25% of cases, while calcifications are rare, usually marginal and dystrophic, probably caused by tumoral microrupture and leak of contents [4]. Detection of enhancing tissue, with or without edema, displaying low signal intensity on DW images should raise the suspicion of malignant transformation [5].

MR Spectroscopy (MRS) of IECs typically show lactate signals at 1.3ppm, with a small peak at 1.8ppm [6]. Few reports exist about the spectral pattern of primary intacranial SCC. Elevation of lactate and absence of NAA peak are expected, while slightly elevated choline/­creatine (Cho/Cr) ratio within the mass [7] and presence of aminoacids [8] have been observed in case reports. In our case the IEC displayed medium lipid/lactate peak, Cho/Cr ratio and NAA/Cho ratio 1,79 and 1,31 respectively, while the component with malignant transformation displayed high lipid/lactate peak, Cho/Cr and NAA/Cho ratio 2,13 and 1,02 respectively. These results are consistent with previous reports describing the tendency of high-grade cystic intacranial tumors toward higher lactate peaks, higher Cho/Cr and lower NAA/Cho ratios [6, 9]. In ­spectral patterns of extracranial primary SCC of the head and neck, the Cho/Cr ratio greater than 1.8 has been reported [10, 11].

Our results suggest that MRS can play a role in the diagnosis of malignant transformation of IECs.

Surgery, radiation therapy, stereotactic therapy and gamma-knife radiosurgery can improve the short-term survival, but long-term prognosis is poor.

## Conclusion

Malignant transformation of an IEC can be suspected by rapid deterioration of symptoms and signs, but MR imaging has a special contribution in diagnosing malignant transformation. Presence of enhancing tissue with low signal intensity on DWI should alert for malignant transformation. Furthermore, MRS can show higher lactate or lipid/lactate peaks, as well as lower NAA/Cho and higher Cho/Cr ratios.

## Competing Interests

The authors declare that they have no competing interests.
